# Proprioceptive measurements of perceived hand position using pointing and verbal localisation tasks

**DOI:** 10.1371/journal.pone.0210911

**Published:** 2019-01-17

**Authors:** Lewis A. Ingram, Annie A. Butler, Simon C. Gandevia, Lee D. Walsh

**Affiliations:** 1 Neuroscience Research Australia, Sydney, New South Wales, Australia; 2 University of New South Wales, Sydney, New South Wales, Australia; 3 Platypus Technical Consultants Pty Ltd, Canberra, Australia; University of Ottawa, CANADA

## Abstract

Previous studies revealed that healthy individuals consistently misjudge the size and shape of their hidden hand during a localisation task. Specifically, they overestimate the width of their hand and underestimate the length of their fingers. This would also imply that the same individuals misjudge the actual location of at least some parts of their hand during the task. Therefore, the primary aim of the current study was to determine whether healthy individuals could accurately locate the actual position of their hand when hidden from view, and whether accuracy depends on the type of localisation task used, the orientation of the hidden hand, and whether the left or right hand is tested. Sixteen healthy right-handed participants performed a hand localisation task that involved both pointing to and verbally indicating the perceived position of landmarks on their hidden hand. Hand position was consistently misjudged as closer to the wrist (proximal bias) and, to a lesser extent, away from the thumb (ulnar bias). The magnitude of these biases depended on the localisation task (pointing vs. verbal), the orientation of the hand (straight vs. rotated), and the hand tested (left vs. right). Furthermore, the proximal location bias increased in size as the duration of the experiment increased, while the magnitude of ulnar bias remained stable through the experiment. Finally, the resultant maps of perceived hand location appear to replicate the previously reported overestimation of hand width and underestimation of finger length. Once again, the magnitude of these distortions is dependent on the task, orientation, and hand tested. These findings underscore the need to control and standardise each component of the hand localisation task in future studies.

## Introduction

Proprioception provides information about the body’s position in space, a fundamental requirement for motor control. While muscle spindles, joint capsules, and the skin provide proprioceptive afferent signals specifying the degree to which each joint is flexed or extended (e.g. ref [1–4]), knowing the spatial configuration of the body is insufficient to determine its position in space. Information about size and shape of the body’s parts is necessary to localise its position (e.g. [5–7]), yet this is not provided by proprioceptive and tactile inputs. This has led researchers to propose the presence of a centrally stored representation of the body’s metric properties referred to as the *body model* [7] or a *body form representation* [8].

Longo & Haggard [9] developed a method to investigate the body model. Sitting with their left hand placed palm down underneath an occluding screen, healthy participants were asked to localise the position of various hand landmarks (the tips and metacarpophalangeal joints of each finger) with a long metal baton (35 cm length and 2 mm diameter) held in their right hand. Images of the hand were created by measuring the judged location of each landmark relative to the others, ignoring any localisation bias in external space. These hand representations were then compared to the actual size and shape of the hand. This revealed a systematic distortion with the fingers shorter and the hand wider than in the real hand. These distortions persisted with the hand rotated 90°, and were also present in the right hand, leading the authors to conclude that the body model for the hand is distorted in a systematic manner.

These methods have subsequently revealed that these distortions of perceived hand dimensions are smaller for the palm of the hand [10], present when blindfolded [11], and altered by changing the posture of the fingers [12]. However, although these distortions were retained in an amputee [13], they were absent during a visual template-matching task [14] (although finger width was overestimated by 32% following an injection of local anaesthesia [15]). Furthermore, these biases may be influenced by perceptual and conceptual distortions [16]. Similar distortions have also been shown when responding to tactile stimuli on the skin surface of the hand [17–19].

While the studies noted above investigated biases in localisation judgements of different landmarks *relative to each other*, the difference between the judged and actual location of each landmark was not reported. Since healthy participants consistently misjudge their hand as shorter and wider than its real size, then the judged position of at least some landmarks relative to their actual location cannot be accurate. Could these distortions in perceived hand shape simply reflect an underlying localisation bias of the hand in external space? If they do, this implies that participants cannot accurately localise the position of the landmarks of their hand. Knowing the size of any baseline mislocalisations is important for understanding proprioception in healthy people. This in turn would allow setting of thresholds that could be used in clinical practice.

Healthy participants consistently misjudge the location of their unseen hand as closer to their wrist (proximal) along the sagittal plane [20–22], and away from the thumb (medial) along the transverse plane [23–31]. The magnitude of these directional biases range from 8mm [20] to 21.5mm [25]–or 1.36° [21] to 3.96° [26]; depending on the units of measurement. Furthermore, the magnitude of error reportedly increases over time [21,22]; a phenomenon referred to as ‘proprioceptive drift.’ However, comparisons across studies are cumbersome due to differences in methodology such as the type of localisation task (pointing vs. verbal judgement), the specific landmark that was localised, the orientation of the hand (straight ahead vs. rotated), the hand tested (left vs. right), different units of measurement (distance vs. angle) and the time taken to perform and complete the localisation task. It is critical to control for these variables if we are to understand how the brain judges the position of the hand in space, and to determine whether these localisation errors underlie the previously reported distortions in perceived hand shape.

Therefore, our study was planned to investigate three specific research questions. Firstly, do healthy participants accurately locate the actual position of various landmarks of their hand, when hidden from view? If so, does the perceived location of their hand remain consistent between pointing (i.e. a motor response involving reaching by the contralateral hand) and verbal (i.e. no limb motor response) judgements, different orientations of the hand (straight vs. rotated positions) and across each hand (left vs. right)? Secondly, does the perceived location of the hand change over the time course of the experiment? Finally, we confirm whether the resultant maps of perceived hand location replicate the previously reported distortions in perceived size and shape of the hand.

## Materials and methods

### Participants

Twenty healthy individuals (10 male, aged 21–49 years) were recruited for the study. The three left-handed participants, as assessed by the Edinburgh Inventory [32], were excluded from analysis. One other participant was excluded on the basis that their hand moved more than 1 cm during each block of trials (see below). Therefore, the study used data from 16 healthy right hand dominant participants (6 male, aged 25–49 years). All subjects provided informed written consent. The study was approved by the Human Research Ethics Committee at the University of New South Wales.

### Procedure

Each participant underwent four separate blocks of trials, each lasting approximately 20 minutes. Each block represented one of four different hand postures–left-hand straight, right-hand straight, left-hand rotated, and right-hand rotated (see below for more detailed description). The order of blocks was randomised across participants. Participants were allowed to take a break between blocks to move their hands.

**Fig 1:**
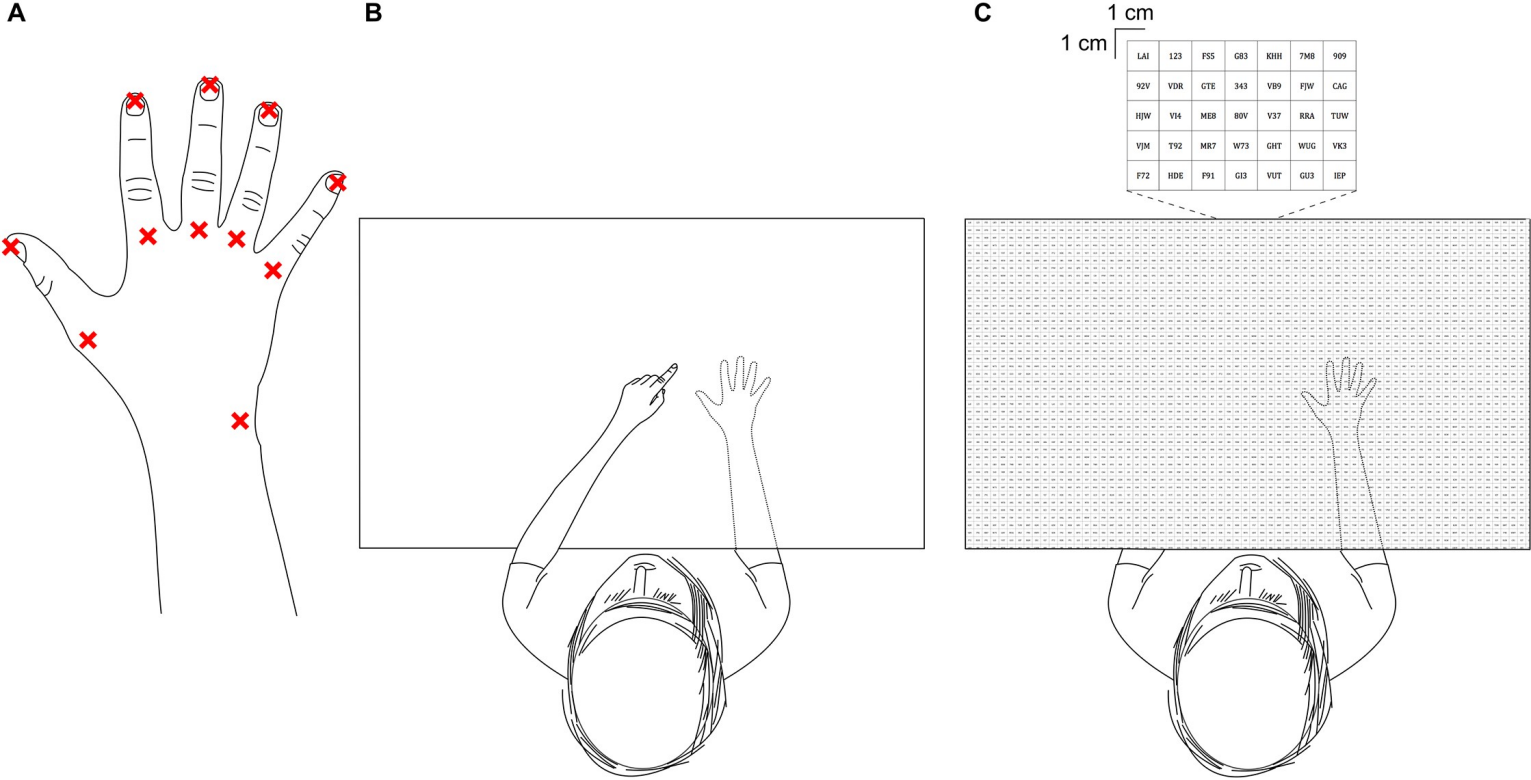
Experimental setup. **(A)** The eleven landmarks of the hand that each participant was asked to locate throughout the experiment. **(B)** A top down view of the participant performing a pointing trial. The participant uses the tip of their contralateral index finger to indicate where they perceive the requested landmark on their test hand (shown in the dashed outline of the hand, which was hidden from the participant’s view beneath a board) is located. **(C)** A top down view of the participant performing a verbal trial. The participant reads aloud the numbers/letters contained within the 1 cm x 1 cm square box which best corresponds to where they perceive the requested landmark on their test hand (which again was hidden from view beneath the same board) is located.

Procedures for the current study were similar to those of Longo & Haggard [9]. Participants sat at a table and were instructed to actively place their hand palm down. Once in position, they were asked to not move their fingers or hand until the end of each block of trials (see below). The table was contained within an experiment booth designed to remove any visual cues from the surrounding environment. The participant’s fingers were gently taped down to prevent movement of the hand. A pen was used to mark eleven separate landmarks on each participant’s hand. These included the tips and metacarpophalangeal joints (knuckles) of each finger, and the styloid process of the ulna (Fig 1A). An overhead camera captured an image of the participant’s marked hand. A blank white screen suspended on four pillars then covered the participant’s hand and forearm. The screen was positioned as close as possible to the back of the participant’s hand and forearm without direct contact (never more than 10 mm from the highest point of the hand). This process took about two minutes. The hand remained covered throughout each block of trials. At the end of each block, the screen was removed and another image was captured with the overhead camera. This was to check that the participant’s hand had not moved throughout the course of the block.

Prior to each trial in each block, pre-recorded verbal instructions were delivered through headphones worn by the participant indicating a particular landmark on the hand (e.g., “tip of the ring finger”), corresponding to one of the 11 landmarks. The pre-recorded verbal instructions and grid projection (see below) were controlled and delivered by purpose-made software (LabBot framework version 0.2, http://tanglo.github.io/LabBot/releases). Participants were then asked to respond by either *pointing* or *verbally*. In the *pointing condition*, participants pointed with the tip of their contralateral index finger to indicate the location of the requested landmark on top of the blank white screen (Fig 1B). Participants said when they were satisfied with their response before the overhead camera captured the position of their contralateral index finger. They were then instructed to return their pointing hand back to their lap prior to the next trial. In the *verbal condition*, an overhead projector displayed a 53 cm x 73 cm square grid onto the blank white screen (Fig 1C). Each square was 1 cm x 1 cm and contained a random sequence of one to three alphanumeric characters. Participants read aloud the characters within the square that best corresponded to the perceived position of the requested landmark, which was recorded by the experimenter. Participants were asked to report if they had mistakenly identified the wrong landmark. These specific responses were noted and removed from subsequent analysis (131 out of 6820, or 1.92% of all trials). Each block consisted of 110 trials (10 per landmark; 5 *pointing condition and 5 verbal condition*) presented in a randomised order.

The hand tested (*left* or *right*) and hand orientation (*straight* or *rotated*) varied by block. In the *straight* blocks, the participant’s forearm was positioned perpendicular to their ipsilateral shoulder such that the fingers were pointing straight ahead. On average, the centre of the actual hand (defined as the mean coordinate values derived from the 11 landmarks of the participant’s actual hand position), when it was straight, was located 34.9 cm (SD = 1.8 cm) in front of each participant (in the sagittal plane) and offset 11.3 cm (SD = 1.1 cm) from their midline (in the transverse plane). In the *rotated* conditions, the initial intention was for the forearm to be placed parallel to the torso so that the radio-ulnar axis of the hand was perpendicular to the participant’s body. However, due to reports of discomfort and occasional mild paraesthesia in the ulnar nerve distribution, this criteria was amended so that the forearm was positioned *approximately* parallel to the body in a comfortable position that the participant could sustain for the duration of the block of trials (approximately 20 minutes). Therefore, the mean angle of the forearm relative to the participants’ torso was 110° (SD = 5.5°). Placement of the wrist with respect to the participant’s midline was not standardised precisely, with the centre of the actual hand offset 12.5 cm (SD = 2.4 cm) from the midline in the contralateral direction (i.e. the rotated left hand was positioned 12.5 cm to the right of the midline). The centre of the actual hand was located 17.9 cm (SD = 2.1 cm) in front of each participant when the hand was rotated.

### Data analysis

#### Construction of hand maps

Before each experimental session, a photo of a setsquare on the experimental table was captured by the overhead camera. The setsquare was used to calculate a calibration curve, which was used to transform the Cartesian coordinate units from the pixel position in the photographs to centimetres (see below). At the start and end of each block of trials, the camera again captured the actual position of each participant’s hand. The Cartesian coordinates of all 11 landmarks for both the ‘before’ and ‘after’ photos were initially measured in pixels using ImageJ [33] before being transformed to centimetres using the aforementioned calibration curve. A mean actual hand position map was then calculated by taking the mean coordinates for each of the 11 landmarks from the ‘before’ and ‘after’ actual hand images. All four blocks of trials from a single participant, along with a single block of trials from a further two participants were excluded from analysis on the basis that their hand had moved > 1 cm during the block.

Coordinates were measured and transformed into cm from the images captured during each of the pointing trials using the same calibration curve outlined above. For verbal responses the coordinates were reported in centimetres from the purpose-made software used to project the grid. Because the coordinates for each verbal response were derived from the bottom left corner of each selected grid box, 0.5 cm was added to each coordinate to centre it in the middle of each grid box.

The coordinates derived from the photos of the participant’s hand were used to create a map of the ‘actual position’ of each landmark. The coordinates derived from the pointing responses were converted into a map of the perceived position of each landmark by taking the mean coordinate positions of the five trials of each individual landmark. The same process was used to create a map of the perceived position of each landmark from the verbal trials. Therefore, 12 hand maps were created for each participant–a mean actual position for each of the four hand conditions (left hand straight, right hand straight, left hand rotated, right hand rotated), along with a perceived position for both the pointing and verbal trials for the same four hand conditions.

To facilitate comparison of data from different subjects, the actual position of the styloid process of the ulna was set to the coordinate 0,0 cm. Each other coordinate for was transformed accordingly.

### Main analysis

To allow comparisons between each orientation (straight and rotated) and each hand (left and right), the abscissa and ordinate axes were transformed to replicate a hand-centred frame of reference. Therefore, the calibrated coordinates calculated previously along the ordinate axis in the straight blocks and the abscissa axis in the rotated blocks now correspond to the proximo-distal axis of the hand (*along* the hand, with positive values representing misjudgements in the proximal direction–i.e. towards the wrist). The calibrated coordinates calculated previously along the abscissa axis in the straight blocks and the ordinate axis in the rotated block now correspond to the radio-ulnar axis of the hand (*across* the hand, with positive values representing misjudgements in the ulnar direction–i.e. away from the thumb).

#### Perceived hand location

For every trial (pointing and verbal), the difference between the perceived location and the actual location of the specific landmark was calculated in cm along both axes. The mean difference between the perceived location and actual location of all 11 landmarks from every pointing and verbal response (i.e. 55 pointing trials, 55 verbal trials) was then calculated to produce a mean pointing perceived hand position and a mean verbal perceived hand position, respectively. Both of these are reported as their difference from actual hand location along both proximo-distal axis of the hand and the radio-ulnar axis of the hand. Therefore, each block resulted in four mean error values; a mean pointing error value along the proximo-distal axis, a mean pointing error value along the radio-ulnar axis, a mean verbal error value along the proximo-distal axis, and a mean verbal error value along the radio-ulnar axis. This procedure was used for each of the four blocks, resulting in 16 mean error values for each participant (8 values along the proximo-distal axis, 8 values along the radio-ulnar axis). Differences between perceived and actual hand location were analysed separately for each axis.

#### Does the perceived location of the hand drift over time?

To determine whether the magnitude and/or direction of any localisation errors remained stable throughout the duration of each block, the mean of each initial and final judgement for every landmark for both the pointing and verbal trials and all of the four hand conditions was calculated.

The initial and final trials were selected on the basis of Wann & Ibrahim’s [22] report that the relationship between drift and time was linear. The difference between the mean overall initial and final judgement of the hand was then determined by taking the mean of each of the 11 landmarks first and final judgement separately for both tasks (pointing and verbal) for each of the four hand conditions (i.e. left straight, right straight, left rotated, right rotated).

When a mistrial occurred during either the first or final trial for a specific landmark, the second or fourth trials were used respectively. This occurred in 60 out of 1420, or 4.23% of all cases. Likewise, each value was divided into a proximo-distal and a radio-ulnar component for further analyses.

#### Size and shape of the perceived hand

Finally, the size and shape of the perceived hand were analysed by calculating the mean finger length and mean hand width for each participant separately for both pointing and verbal trials for each of the four blocks. Finger length was calculated as the mean distance between the tip and knuckle (metacarpophalangeal joint) of each digit in cm, while hand width was taken as the distance between the knuckles (metacarpophalangeal joints) of the index and little finger in cm. Both perceived finger length and hand width were calculated and are reported as a percentage of actual finger length and actual hand width for each combination of task, orientation and hand. This was done to take into account differences in hand size between participants.

### Statistical analysis

Each of the research questions (see above) was analysed and compared across each combination of task, orientation and hand using mixed linear models [34,35]. For both the mislocation of hand position and drift of perceived hand position, a separate analysis was performed for both proximal error and ulnar error. Perceived finger length and perceived hand width were each analysed with a single model. Therefore, a total of six separate mixed linear model analyses were performed. Each analysis was initially specified as a second-order interaction in a hierarchical model containing three fixed factors (task [2 levels–pointing vs. verbal], orientation [2 levels–straight vs. rotated], and hand [2 levels–left vs. right]) and their interactions (task × orientation, task × hand, orientation × hand, task × orientation × hand) and a random intercept. If the three-way interaction term was not significant, a first-order interaction model was then run containing all three two-way interactions, along with fixed effects and a random intercept. Likewise, in the absence of any significant two-way interactions, all consecutive interaction terms were removed from the model and the analysis was repeated with fixed effects only and a random intercept. The results of the initial second-order hierarchical interaction model and the final model are reported (Table 1) ([S1 File] contains the results of each interaction model and each fixed effects only model for each analysis). Summary statistics for the fitted models are reported as estimated marginal means. The confidence intervals for any reported mean differences between tasks, orientations and hands reflect the pairwise calculated differences. Significance for all statistical tests was set at *p* < 0.05. All statistical tests were performed using SPSS Statistics Version 25 (IBM, Armonk, NY, USA).

**Table 1:** The results of each second order hierarchical interaction models and each fixed effects only models for each analysis.

	Second-order	*b*	95% CI	*p*	Fixed effects	*b*	95% CI	*p*
**Proximal mislocation**	Intercept (*b*_0_)	1.63	-0.08, 3.33	0.061	Intercept (*b*_0_)	2.21	0.85, 3.57	0.002
	Task (*b*_1_)	-0.62	-2.68, 1.45	0.555	Task (*b*_1_)	-1.18	-2.19, -0.16	0.024
	Orientation (*b*_2_)	4.04	2.00, 6.08	< .001	Orientation (*b*_2_)	2.88	1.85, 3.90	< .001
	Hand (*b*_3_)	1.33	-0.75, 3.40	0.207	Hand (*b*_3_)	1.05	0.03, 2.07	0.044
	Task*Orientation (*b*_4_)	-1.17	-4.05, 1.70	0.420				
	Task*Hand (*b*_5_)	0.64	-2.28, 3.56	0.663				
	Orientation*Hand (*b*_6_)	-0.62	-3.50, 2.26	0.670				
	Task*Orientation*Hand (*b*_7_)	-1.07	-5.14, 3.00	0.604				
**Ulnar mislocation**	Intercept (*b*_0_)	-1.23	-2.65, 0.19	0.089	Intercept (*b*_0_)	-1.16	-2.33, 0.02	0.054
	Task (*b*_1_)	0.92	-0.69, 2.54	0.260	Task (*b*_1_)	0.90	0.11, 1.69	0.026
	Orientation (*b*_2_)	2.98	1.38, 4.57	< .001	Orientation (*b*_2_)	2.29	1.50, 3.09	< .001
	Hand (*b*_3_)	0.88	-0.73, 2.51	0.281	Hand (*b*_3_)	1.18	0.38, 1.97	0.004
	Task*Orientation (*b*_4_)	-1.11	-3.36, 1.14	0.330				
	Task*Hand (*b*_5_)	0.85	-1.43, 3.13	0.463				
	Orientation*Hand (*b*_6_)	-0.51	-2.76, 1.75	0.657				
	Task*Orientation*Hand (*b*_7_)	0.50	-2.67, 3.68	0.755				
**Proximal drift**	Intercept (*b*_0_)	2.16	1.11, 3.20	< .001	Intercept (*b*_0_)	2.47	1.58, 3.36	< .001
	Task (*b*_1_)	0.41	-0.70, 1.52	0.468	Task (*b*_1_)	-0.22	-0.76, 0.32	0.422
	Orientation (*b*_2_)	-0.12	-1.22, 0.98	0.830	Orientation (*b*_2_)	-0.47	-1.02, 0.07	0.089
	Hand (*b*_3_)	0.40	-0.72, 1.52	0.480	Hand (*b*_3_)	0.07	-0.48, 0.61	0.806
	Task*Orientation (*b*_4_)	-0.71	-2.26, 0.84	0.364				
	Task*Hand (*b*_5_)	-0.67	-2.24, 0.90	0.401				
	Orientation*Hand (*b*_6_)	-0.14	-1.69, 1.41	0.861				
	Task*Orientation*Hand (*b*_7_)	0.29	-1.90, 2.48	0.796				
**Ulnar drift**	Intercept (*b*_0_)	-0.28	-1.09, 0.54	0.504	Intercept (*b*_0_)	-0.14	-0.73, 0.44	0.625
	Task (*b*_1_)	-0.16	-1.29, 0.98	0.784	Task (*b*_1_)	-0.12	-0.68, 0.43	0.666
	Orientation (*b*_2_)	1.08	-0.03, 2.20	0.057	Orientation (*b*_2_)	0.69	0.12, 1.24	0.016
	Hand (*b*_3_)	-0.42	-1.55, 0.72	0.470	Hand (*b*_3_)	-0.38	-0.93, 0.18	0.183
	Task*Orientation (*b*_4_)	-0.21	-1.80, 1.37	0.788				
	Task*Hand (*b*_5_)	0.68	-0.93, 2.29	0.403				
	Orientation*Hand (*b*_6_)	-0.20	-1.79, 1.38	0.799				
	Task*Orientation*Hand (*b*_7_)	-0.75	-2.98, 1.49	0.508				
**Finger length**	Intercept (*b*_0_)	-36.92	-45.88, -27.96	< .001	Intercept (*b*_0_)	-34.04	-41.66, -26.42	< .001
	Task (*b*_1_)	12.09	2.43, 21.76	0.015	Task (*b*_1_)	9.64	4.93, 14.35	< .001
	Orientation (*b*_2_)	-5.05	-14.58, 4.48	0.296	Orientation (*b*_2_)	-9.61	-14.34, -4.87	< .001
	Hand (*b*_3_)	9.42	-0.28, 19.13	0.057	Hand (*b*_3_)	3.25	-1.48, 7.99	0.176
	Task*Orientation (*b*_4_)	-2.75	-16.20, 10.70	0.686				
	Task*Hand (*b*_5_)	-5.71	-19.37, 7.96	0.409				
	Orientation*Hand (*b*_6_)	-9.89	-23.37, 3.60	0.149				
	Task*Orientation*Hand (*b*_7_)	7.06	-11.97, 26.08	0.464				
**Hand width**	Intercept (*b*_0_)	15.41	5.11, 35.93	0.137	Intercept (*b*_0_)	12.70	-5.68, 31.08	0.167
	Task (*b*_1_)	29.89	10.99, 48.79	0.002	Task (*b*_1_)	31.99	22.74, 41.25	< .001
	Orientation (*b*_2_)	18.79	0.15, 37.44	0.048	Orientation (*b*_2_)	19.55	10.25, 28.85	< .001
	Hand (*b*_3_)	10.14	-8.86, 29.13	0.292	Hand (*b*_3_)	12.52	3.22, 21.82	0.009
	Task*Orientation (*b*_4_)	-4.84	-31.15, 21.47	0.716				
	Task*Hand (*b*_5_)	-1.86	-28.59, 24.87	0.891				
	Orientation*Hand (*b*_6_)	-4.36	-30.73, 22.02	0.744				
	Task*Orientation*Hand (*b*_7_)	21.43	-15.78, 58.64	0.256				

Note: coding used for variables: task (pointing = 0, verbal = 1), orientation (straight = 0, rotated = 1), and hand (left = 0, right = 1).

## Results

**Fig 2:**
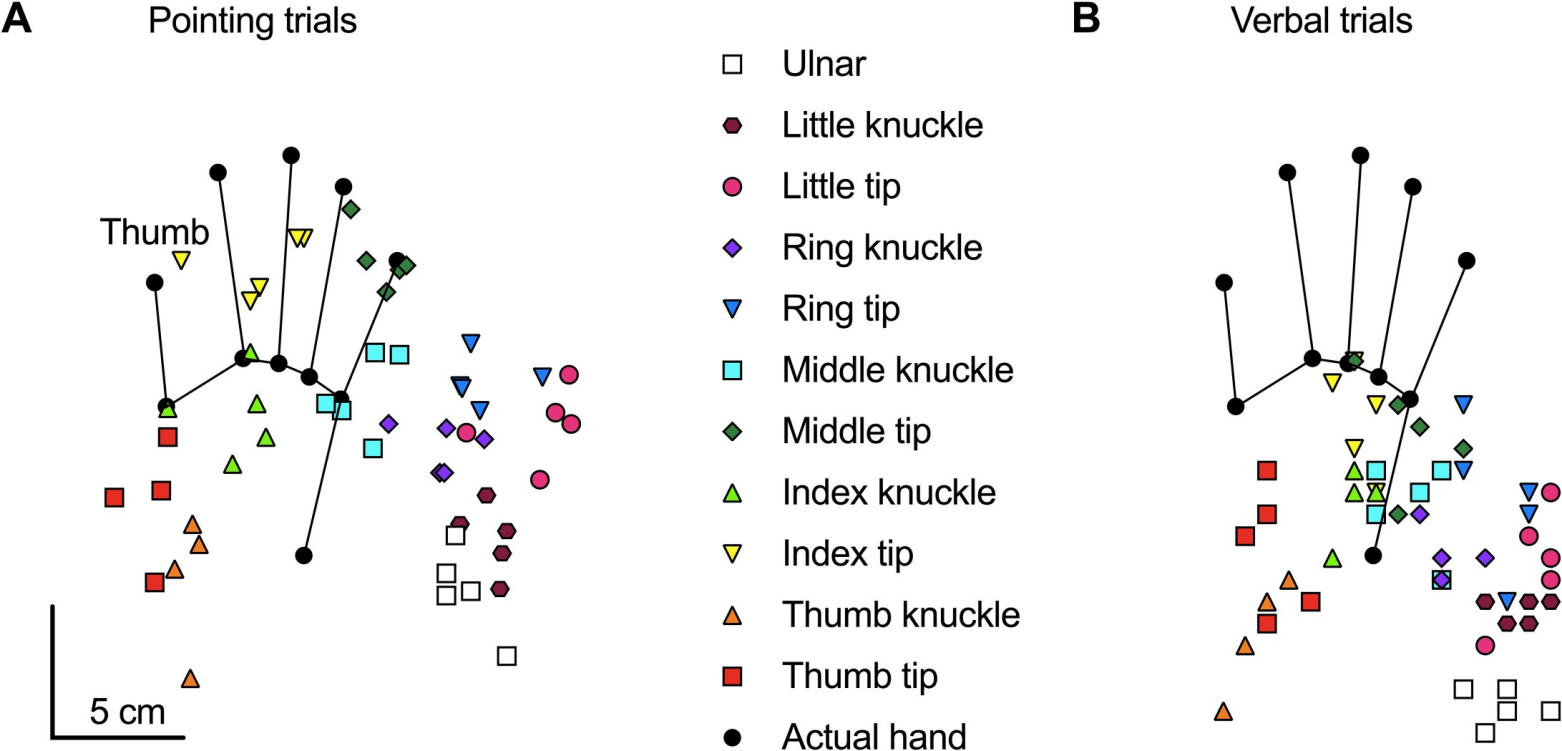
Example participant. Raw data showing all of the pointing responses **(A)** and all of the verbal responses **(B)** from a single block of trials (55 pointing trials, 55 verbal trials) from a single participant performed with the right-hand positioned straight ahead with the palm down. The black circles and connected lines show the actual location of each of the 11 landmarks for this individual participant. The coloured symbols show the location of each individual pointing trial throughout the block. Each specific colour and shape correspond to a specific landmark, as indicated in the middle of the figure.

All of the pointing trials and all of the verbal trials from a single participant performed with their right hand positioned straight are shown in Fig 2A and Fig 2B respectively. During the pointing trials, the participant consistently misjudged their hand as closer to their wrist (proximal error) and away from their thumb (ulnar shift). During the verbal trials, the magnitude of proximal error appears greater while the degree of ulnar mislocation appears smaller. A shortening of finger length and a widening of space between knuckles are also evident in the figure during the pointing trials, while only an apparent shortening of finger length appears during the verbal trials.

**Fig 3:**
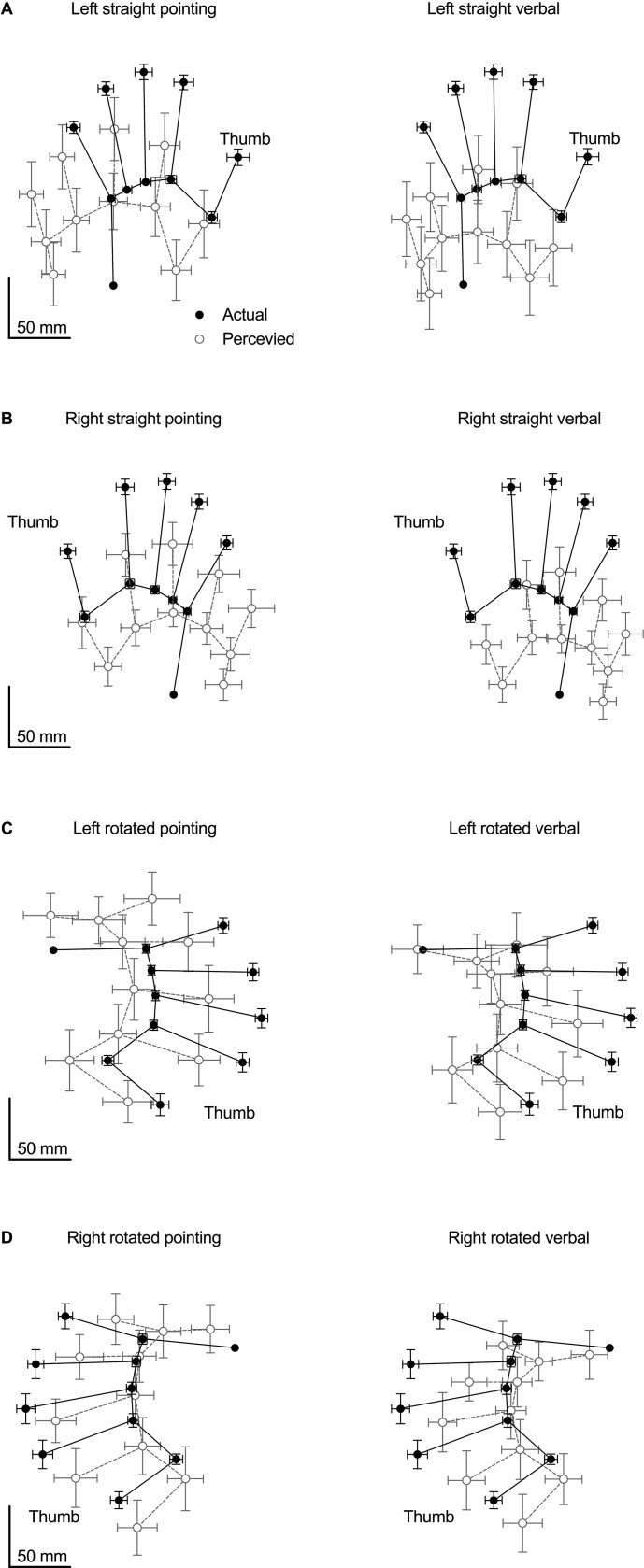
Pooled data. The mean position of all 11-landmark pointing and verbal localisation judgements (shown separately) compared to the mean actual location of each landmark in each of the four hand orientations. The black circles show the mean actual location of each of the 11 landmarks. The unfilled grey circles show the mean judged location of each of the 11 landmarks. Error bars depict 95% confidence intervals. **(A)** Pointing vs. verbal responses when the left hand is straight. **(B)** Pointing vs. verbal responses when the right hand is straight. **(C)** Pointing vs. verbal responses when the left hand is rotated. **(D)** Pointing vs. verbal responses when the right hand is rotated.

The mean judged location for each landmark, calculated from all 16 participants, compared to its actual location is shown in Fig 3. Most conditions demonstrate a shift in both the proximal and ulnar directions, along with a shortening of finger length and widening of hand width.

### Proximal mislocation

**Fig 4:**
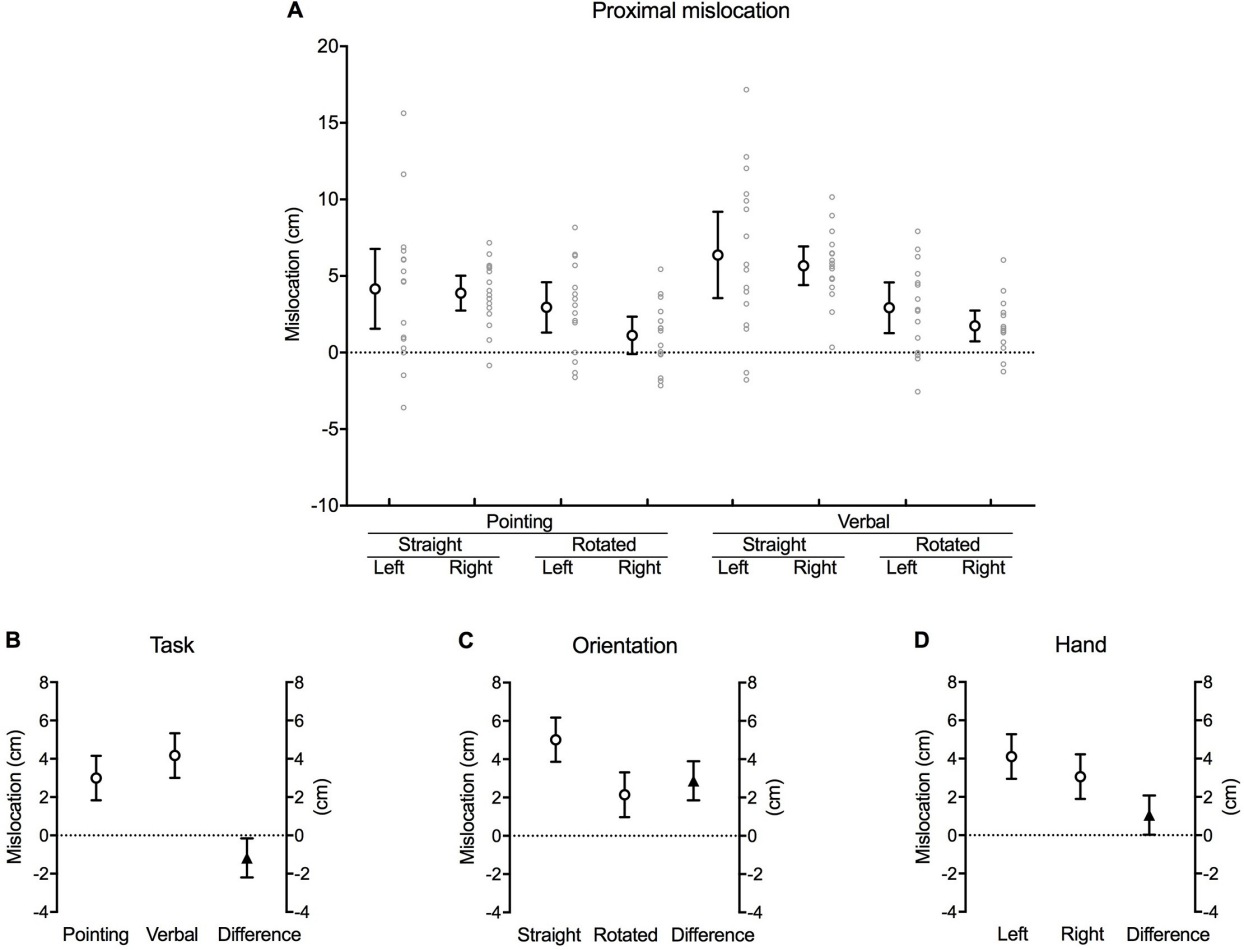
Proximal mislocation. **(A)** Difference between the actual and perceived hand location (cm) along the proximo-distal axis of the hand for each combination of task, orientation and hand (large white circles with error bars, which depict 95% confidence intervals). Adjacent small grey circles represent individual data points. Positive values represent mislocation in the proximal direction (*towards* the wrist). **(B)** The difference between pointing and verbal localisation tasks, **(C)** straight and rotated hand orientations, and **(D)** the left and right hand after the data were fitted to a fixed effects with a random intercept model (see Materials and Methods). The white circles in each graph show the estimated marginal mean values for each factor within each main effect. The black triangles show the mean difference between each factor (this value corresponds to its respective *b* coefficient). Error bars depict 95% confidence intervals.

Participants mislocated the actual position of their hand in the proximal direction for each combination of task, orientation and hand, except for when they pointed to their right hand when it was rotated (Fig 4A). This is indicated by the 95% confidence interval error bars crossing zero during the right rotated pointing condition.

As there was no significant three-way interaction in the initial second-order interaction hierarchical model, the analysis was rerun with first-order interaction terms. Again, there were no significant two-way interactions, so all consecutive interaction terms were removed and the analysis was rerun with a model consisting of fixed effects only and a random intercept (Table 1). This was the also the case in each of the subsequent analyses. Therefore, all of the reported main effects in the following sections were derived from models consisting of fixed effects only and a random intercept.

There was a significant main effect of task [*b* = –1.18, *t*(–2.289), *p* = 0.024], orientation [*b* = 2.88, *t*(5.576), *p* < 0.001] and hand [*b* = 1.05, *t*(2.037), *p* = 0.044]. Inspection of the estimated marginal means of the fitted model reveals that participants were more accurate at locating the position of their hidden hand when pointing with their contralateral hand (3.00 [1.83, 4.16] cm proximally from the actual location of their hand) (mean [95% confidence interval]) compared to responding verbally (4.17 [3.01, 5.33] cm) [mean difference –1.18 [–2.19, –0.16] cm, *p* = 0.024] (Fig 4B). Likewise, the magnitude of proximal error was greater when the hidden hand was positioned straight (5.02 [3.86, 6.18] cm) compared to rotated (2.15 [0.97, 3.32] cm) [mean difference 2.88 [1.85, 3.90] cm, *p* < 0.001] (Fig 4C). Lastly, participants were less accurate when judging the position of their left (non-dominant) hand (4.11 [2.94, 5.27] cm) compared to their right (dominant) hand (3.06 [1.89, 4.22] cm) [mean difference 1.05 [0.03, 2.07] cm, *p* = 0.044] (Fig 4D).

### Ulnar mislocation

**Fig 5:**
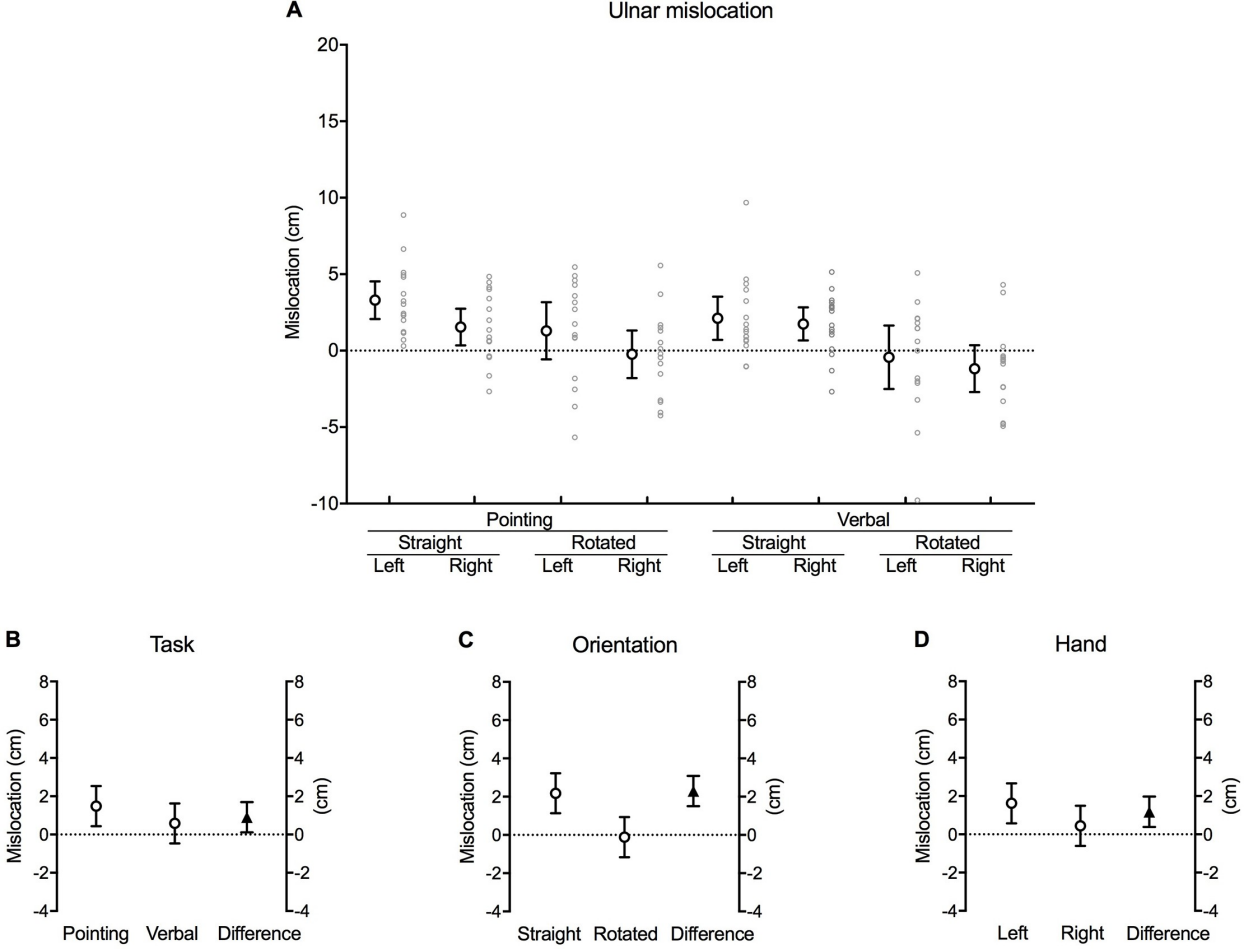
Ulnar mislocation. **(A)** Difference between the actual and perceived hand location (cm) along the radio-ulnar axis of the hand for each combination of task, orientation and hand (large white circles with error bars, which depict 95% confidence intervals). Adjacent small grey circles represent individual data points. Positive values represent mislocation in the ulnar direction (*away* from the thumb). **(B)** The difference between pointing and verbal localisation tasks, **(C)** straight and rotated hand orientations, and **(D)** the left and right hand after the data were fitted to a fixed effects with a random intercept model. The white circles in each graph show the estimated marginal mean values for each factor within each main effect. The black triangles show the mean difference between each factor (this value corresponds to its respective *b* coefficient). Error bars depict 95% confidence intervals.

Participants mislocated their actual hand position as further away from their thumb (ulnar error) during all of the conditions when the test hand was orientated straight (Fig 5A). However, in contrast to the proximo-distal axis, that there was no robust difference in actual and perceived hand position along the radio-ulnar axis when the hand was rotated, as indicated by the 95% confidence interval error bars for each of the rotated conditions crossing zero.

There was a significant main effect of task [*b* = 0.90, *t*(2.265), *p* = 0.026], orientation [*b* = 2.29, *t*(5.727), *p* < 0.001] and hand [*b* = 1.18, *t*(2.941), *p* = 0.004] (Table 1). Investigating the estimated marginal means of the fitted model suggests that participants were, on average, accurate when locating the position of their hidden hand along the radio-ulnar axis during the verbal task (0.58 [–0.48, 1.63] cm), shown by the 95% confidence interval crossing zero. Conversely, pointing resulted in a mean 1.48 (0.44, 2.53) cm error in the ulnar direction (*away* from the thumb) [mean difference 0.90 [0.11, 1.69] cm, *p* = 0.026] (Fig 5B). Similarly, estimations of hand position were, on average, accurate when the hidden hand was rotated (–0.12 [–1.17, 0.94] cm) compared with the mean 2.18 (1.14, 3.22) cm error in the ulnar direction when the hand was straight [mean difference 2.29 [1.50, 3.09] cm, *p* < 0.001] (Fig 5C). Lastly, participants were, on average, accurate when judging the position of their right (dominant) hand (0.44 [–0.61, 1.49] cm), while errors persisted in the ulnar direction at the left (non-dominant) hand (1.62 [0.57. 2.67] cm) [mean difference 1.18 [0.38, 1.97] cm, *p* = 0.004] (Fig 5D).

### Proximal drift

**Fig 6:**
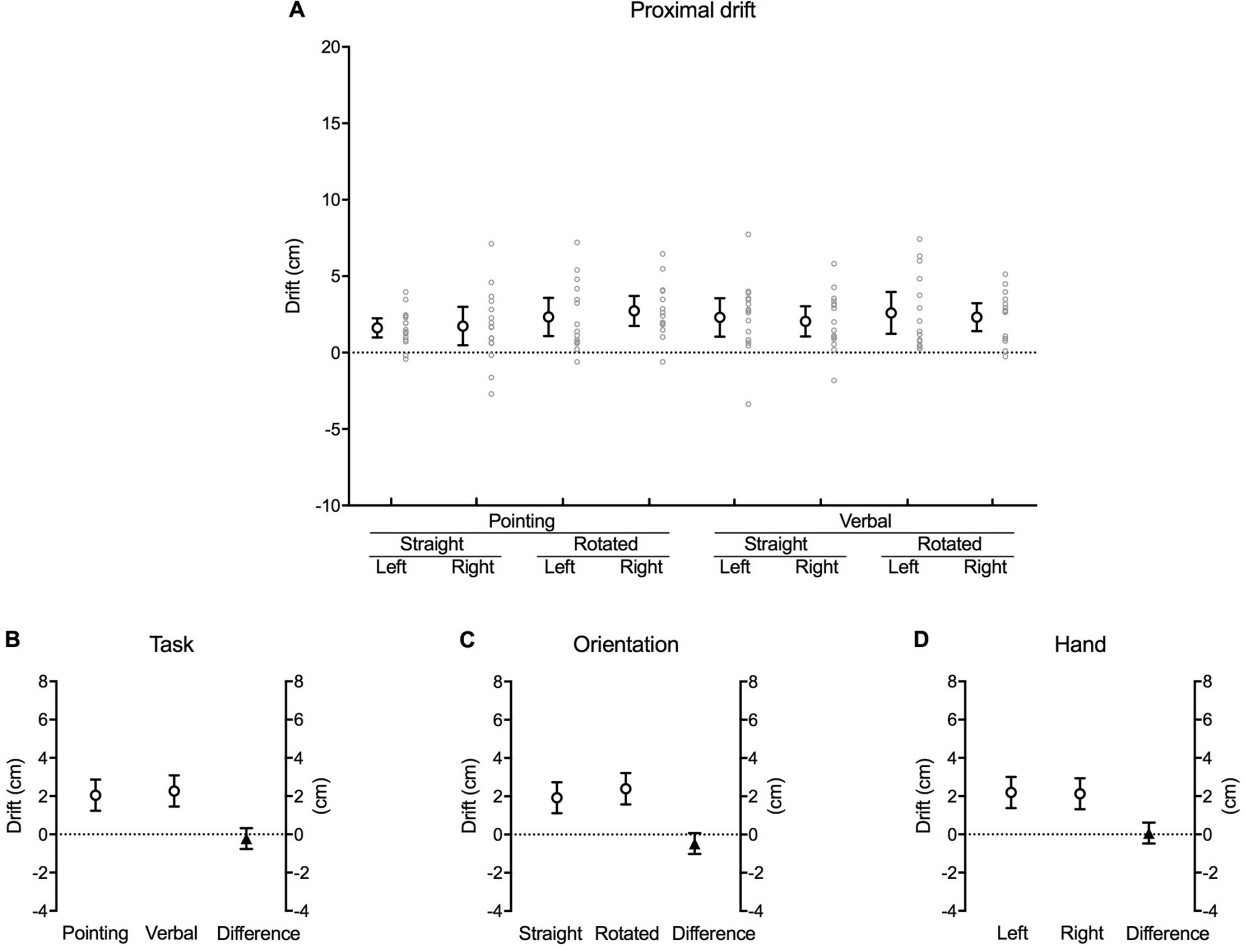
Proximal drift. **(A)** Mean difference (cm) in perceived hand position between the first and last trial along the proximo-distal axis for each combination of task, orientation and hand (large white circles with error bars, which depict 95% confidence intervals). Adjacent small grey circles represent individual data points. Positive values represent a drift in perceived hand position in the proximal direction (*towards* the wrist). **(B)** The difference between pointing and verbal localisation tasks, **(C)** straight and rotated hand orientations, and **(D)** the left and right hand after the data were fitted to a fixed effects with a random intercept model. The white circles in each graph show the estimated marginal mean values for each factor within each main effect. The black triangles show the mean difference between each factor (this value corresponds to its respective *b* coefficient). Error bars depict 95% confidence intervals.

The actual position of the hand was progressively misjudged as closer towards the wrist (in the proximal direction) over the time course of each block of trials (Fig 6A). This occurred irrespective of task, orientation and hand, illustrated by the fact that none of the 95% confidence interval error bars cross zero.

There were no main effects for task [*b* = –0.22, *t*(–0.806), *p* = 0.422] (Fig 6B), orientation [*b* = –0.47, *t*(–1.719), *p* = 0.089] (Fig 6C) or hand [*b* = 0.07, *t*(0.246), *p* = 0.806] (Fig 6D) (Table 1).

### Ulnar drift

**Fig 7:**
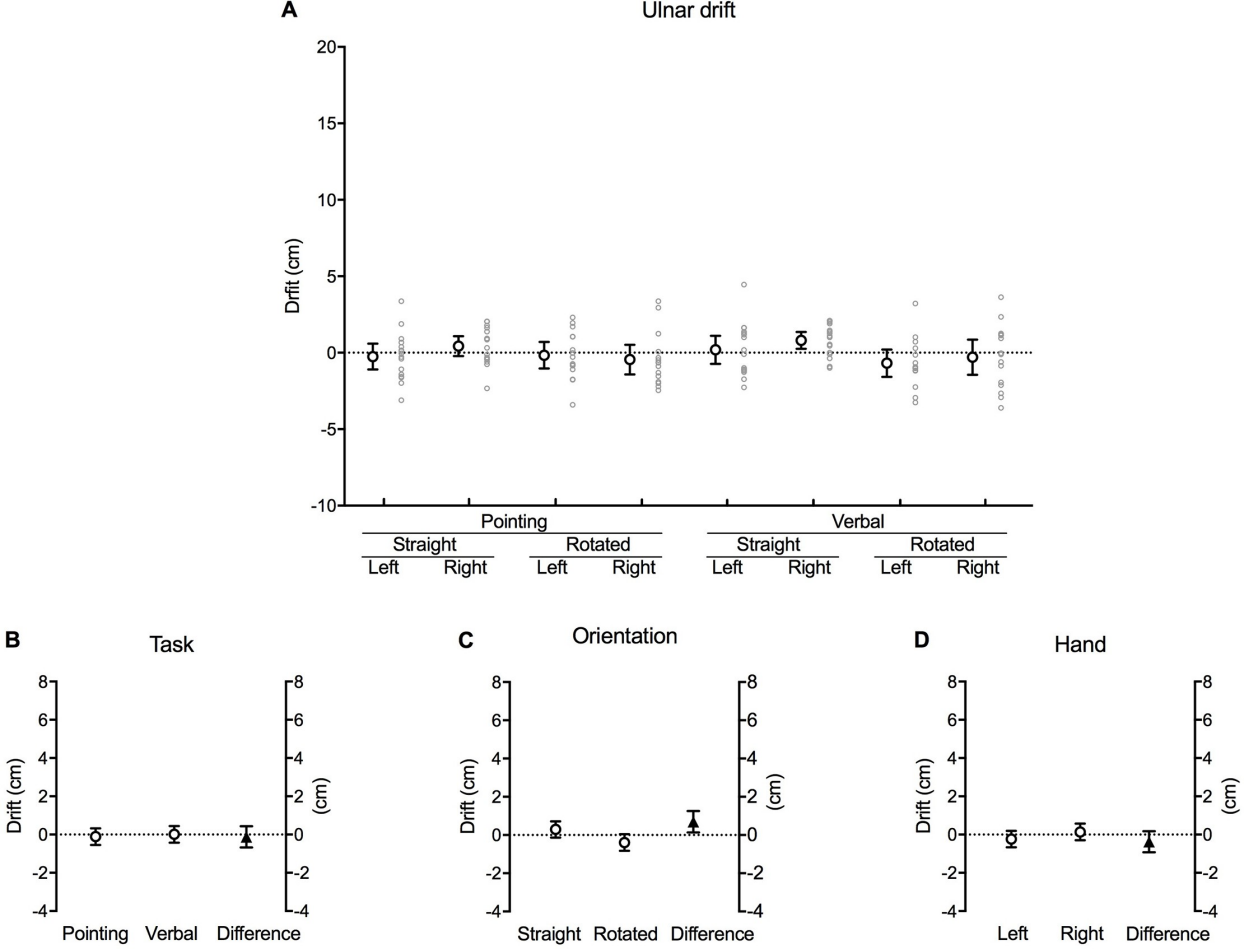
Ulnar drift. **(A)** Mean difference (cm) in perceived hand position between the first and last trial along the radio-ulnar axis for each combination of task, orientation and hand (large white circles with error bars, which depict 95% confidence intervals). Adjacent small grey circles represent individual data points. Positive values represent a drift in perceived hand position in the ulnar direction (*away* from the thumb). **(B)** The difference between pointing and verbal localisation tasks, **(C)** straight and rotated hand orientations, and **(D)** the left and right hand after the data were fitted to a fixed effects with a random intercept model. The white circles in each graph show the estimated marginal mean values for each factor within each main effect. The black triangles show the mean difference between each factor (this value corresponds to its respective *b* coefficient). Error bars depict 95% confidence intervals.

In contrast to the pattern observed for drift in the proximal direction, there was no change in magnitude of mislocation error along the radio-ulnar axis of the hand during each block of trials (Fig 7A), with the exception of verbal responses when the right hand was straight. This is shown with the 95% confidence interval error bars crossing zero except for the verbal trials when the right hand was straight.

There was a significant main effect for orientation [*b* = 0.69, *t*(2.451), *p* = 0.016] (Table 1), once again with the magnitude of the drift appearing greater in the rotated hand (–0.39 [–0.83, 0.05] cm) compared to the straight hand (0.29 [–0.13, 0.72] cm) [mean difference 0.69 [0.13, 1.24] cm, *p* = 0.016] (Fig 7C). Furthermore, the negative sign of the rotated value suggests a small but significant drift in the radial direction (*towards* the thumb). However, there was no main effect for task [*b* = –0.12, *t*(–0.433), *p* = 0.666] (Fig 7B) or hand [*b* = –0.38, *t*(–1.341), *p* = 0.183] (Fig 7D).

### Finger length

**Fig 8:**
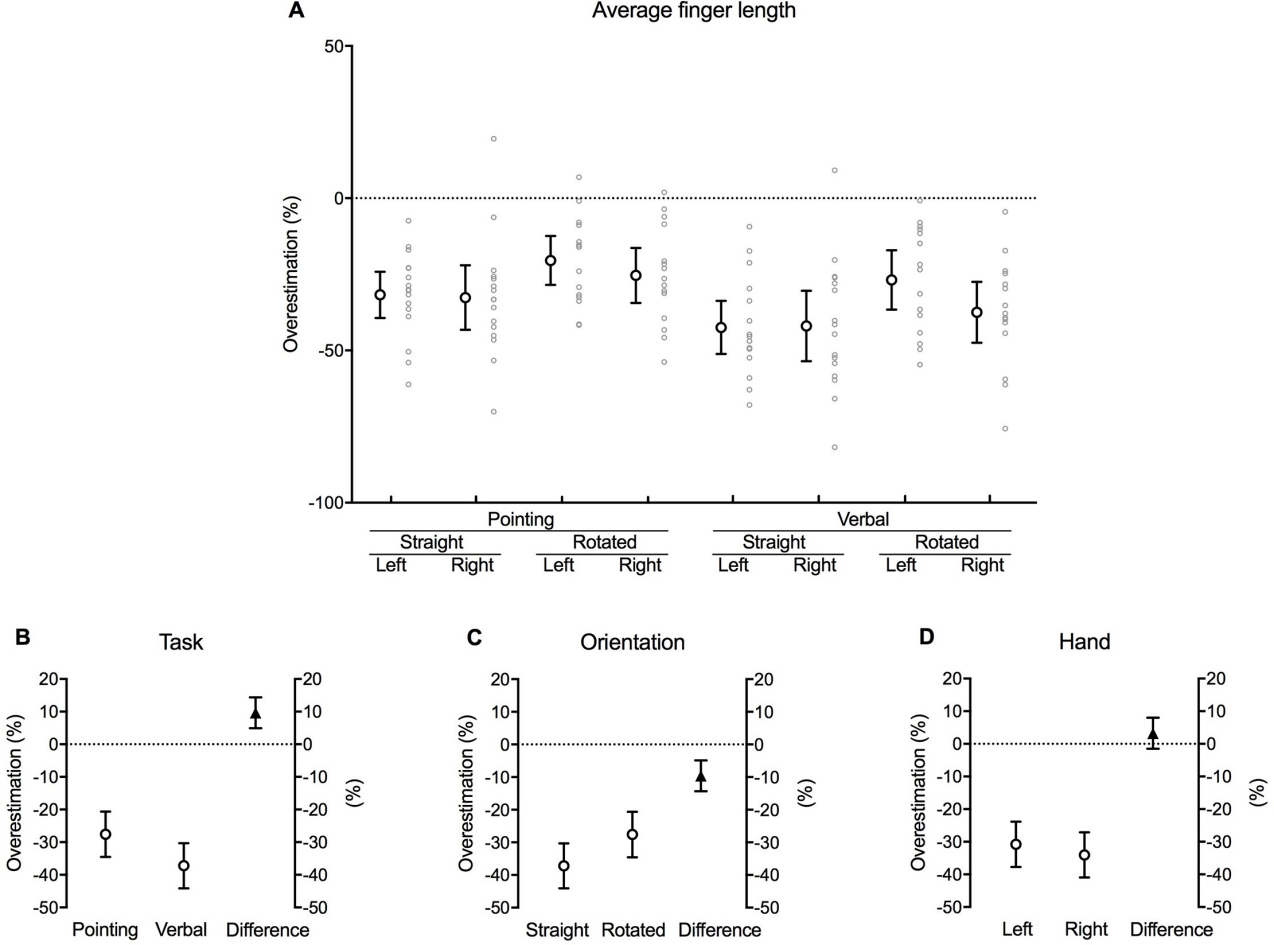
Finger length. **(A)** Mean difference between actual and perceived length of the five fingers for each combination of task, orientation and hand as a percentage of actual finger length (large white circles with error bars, which depict 95% confidence intervals). Adjacent small grey circles represent individual data points. **(B)** The difference between pointing and verbal localisation tasks, **(C)** straight and rotated hand orientations, and **(D)** the left and right hand after the data were fitted to a fixed effects with a random intercept model. The white circles in each graph show the estimated marginal mean values for each factor within each main effect. The black triangles show the mean difference between each factor (this value corresponds to its respective *b* coefficient). Error bars depict 95% confidence intervals.

Perceived finger length–as derived from the landmark judgements–was underestimated compared to actual finger length in each of the conditions (Fig 8A). This is implied by the consistent negative values and with none of the 95% confidence interval error bars crossing zero.

There was a significant main effect of task [*b* = 9.64, *t*(4.056), *p* < 0.001] and orientation [*b* = –9.61, *t*(–4.023), *p* < 0.001] (Table 1). Inspection of the estimated marginal means of the fitted model reveals that the calculated length of the subjects’ fingers was underestimated by a larger degree during the verbal task (–37.22 [–44.14, –30.29] % of actual finger length) compared to pointing (–27.57 [–34.50, –20.65] % of actual finger length) [mean difference 9.46 [4.93, 14.35] % of actual finger length, *p* < 0.001] (Fig 8B). Similarly, the calculated finger length underestimated the actual finger length to a greater extent when the hidden hand was straight (–37.20 [–44.10, –30.30] % of actual finger length) compared to when it was rotated (–27.59 [–34.54, –20.64] % of actual finger length) [mean difference –9.61 [–14.34, –4.87] % of actual finger length, *p* < 0.001] (Fig 8C). There was no effect of hand [*b* = 3.25, *t*(1.362), *p* = 0.176] (Fig 8D).

### Hand width

With the exception of verbal responses when the right hand was rotated, perceived hand width–as derived from the landmark judgments–was consistently overestimated in all remaining conditions (Fig 8A). This is indicated by the consistent positive values, and with none of the 95% confidence interval error bars crossing zero except for the verbal trials when the right hand was rotated.

**Fig 9:**
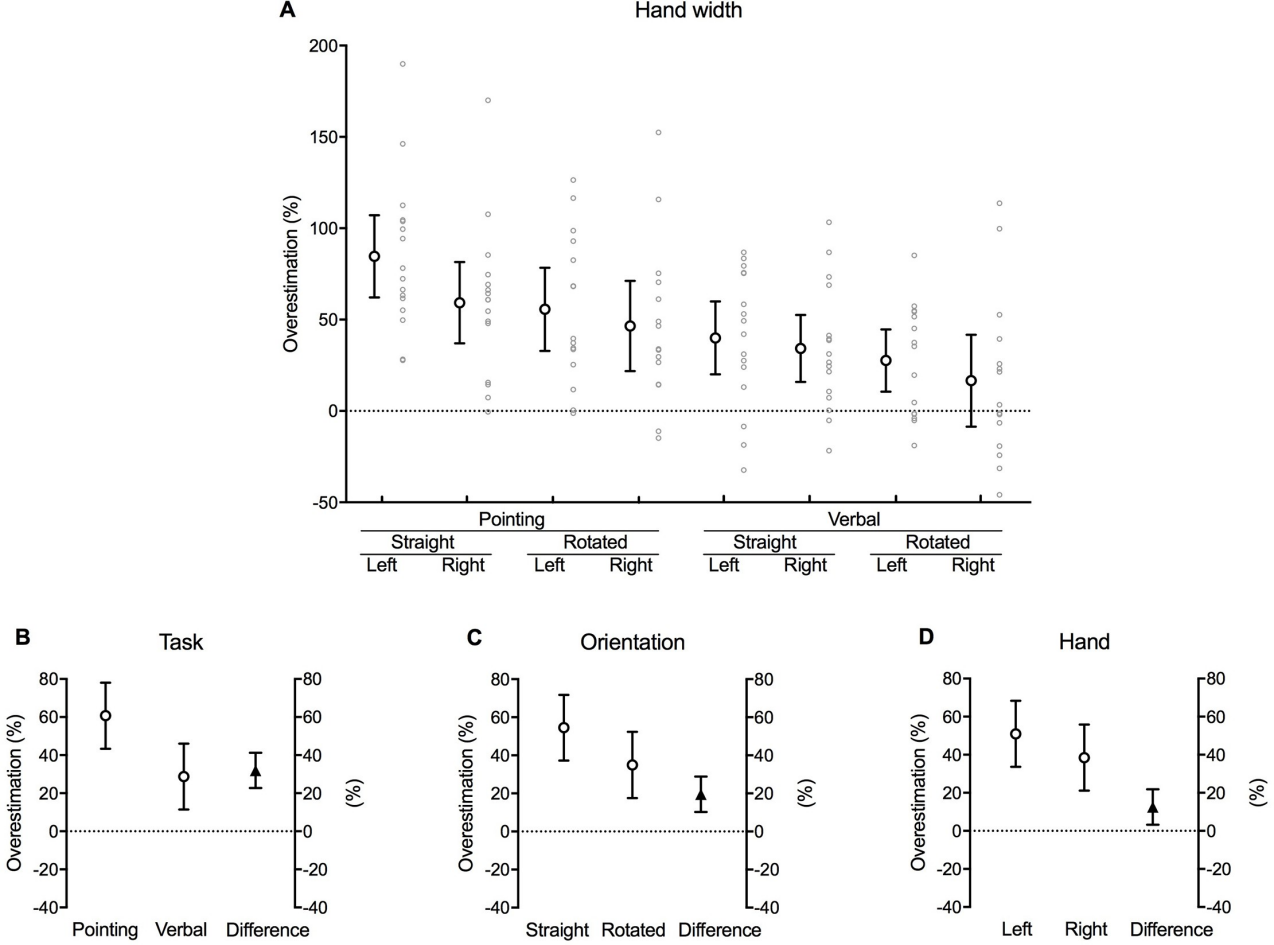
Hand width. **(A)** Mean difference between actual and perceived hand width for each combination of task, orientation and hand as a percentage of the actual distance between the knuckles of the index and little finger (i.e. the width of the hand) (large white circles with error bars, which depict 95% confidence intervals). Adjacent small grey circles represent individual data points. **(B)** The difference between pointing and verbal localisation tasks, **(C)** straight and rotated hand orientations, and **(D)** the left and right hand after the data were fitted to a fixed effects with a random intercept model. The white circles in each graph show the estimated marginal mean values for each factor within each main effect. The black triangles show the mean difference between each factor (this value corresponds to its respective *b* coefficient). Error bars depict 95% confidence intervals.

Significant main effects were observed for task [*b* = 31.99, *t*(6.854), *p* < 0.001], orientation [*b* = 19.55, *t*(4.169), *p* < 0.001] and hand [*b* = –12.52, *t*(2.670), *p* = 0.009] (Table 1). Looking at the estimated marginal means suggests that participants overestimated the width of their hand to a greater degree when pointing (60.73 [43.40, 78.06] % of the width of the hand) compared to that of the verbal localisation task (28.74 [11.41, 46.06] % of the width of the hand) [mean difference 31.99 [22.74, 41.25] % of the width of the hand, *p* < 0.001] (Fig 9B). Similarly, hand width was overestimated to an even greater extent when the hidden hand was straight (54.51 [37.22, 71.80] % of the width of the hand) compared to rotated (34.96 [17.58, 52.33] % of the width of the hand) [mean difference 19.55 [10.25, 28.85] % of the width of the hand, *p* < 0.001] (Fig 9C). Finally, participants tended to overestimate the width of their left (non-dominant) hand (50.99 [33.66, 68.32] % of the width of the hand) more than their right (dominant) hand (38.47 [21.14, 55.80] % of the width of the hand) [mean difference 12.52 [3.22, 21.82] % of the width of the hand, *p* = 0.009] (Fig 9D).

## Discussion

The results of the current study reveal that healthy participants consistently misjudge the location of their hand as closer towards their wrist (proximal bias) compared to its actual location. Although the magnitude of this proximal bias was dependent on the type of localisation task performed and the orientation of the tested hand, some participants mislocated their hand by over 6 cm. Furthermore, this bias in the proximal direction was shown to increase by approximately 2–3 cm over time (proximal proprioceptive drift). In contrast, a smaller and less consistent bias was observed along the radio-ulnar axis of the hand. Likewise, the size of these mislocations changed with the task performed, the orientation of the hand, as well as the hand tested (dominant vs. non-dominant). There was no change in perceived hand location in the radial or ulnar direction over time. The distortions in hand shape and size observed in the resulting maps of perceived hand location (Fig 3) replicate previous findings [9–14,16–19,36–37]. These findings further advance our understanding of human proprioception and build the foundation towards a potential measurement of proprioception in the clinical setting.

### Mislocation of hand position

Although a proximal bias was observed across *most* conditions, it was most apparent both during the verbal localisation task and when the hidden hand was orientated straight in front of the body. In contrast, a small bias of up to 3 cm *away* from the thumb (ulnar bias) was observed *only* when participants pointed to their hidden hand, when their tested hand was straight, and when the hand tested was their left (non-dominant) hand. Verbally indicating the location of the hand, rotating the hand, and testing the right (dominant) hand all resulted in accurate localisation along the radio-ulnar axis.

The directional consistency in proximal mislocation between the straight and rotated hand implies that participants are referencing their judgments of perceived hand position from their hand, rather than from their body or external space. While rotating the hand removes the ulnar bias, there is no clear increase in radial bias that would otherwise be expected when rotating the hand if judgments of perceived hand position were made from the reference frame of the body or external space. These findings suggest that we perceive our hand as closer towards our wrist rather than as closer towards our body, underscoring the importance of the body parts immediately proximal to the region in question in determining its actual location. However, it must be noted that in the current study we were unable to standardise the position of the participant’s hand with respect to their body during the rotated condition. Despite our intentions, aligning the radio-ulnar axis of the participant’s hand with their midline proved slightly uncomfortable for a number of participants to sustain for the duration of each block of trials.

The difference in the size of the bias between the pointing and verbal localisation tasks, along with the reversal of the magnitude between the proximo-distal and radio-ulnar axes raises the question as to whether they are measuring the same construct. The physical action of reaching and pointing initially requires accurate information about the starting position of the contralateral hand; it then generates a central motor command prior to performance, while permitting additional proprioceptive cues throughout the movement [3–4,26,31,38]. In contrast, the verbal localisation task relies solely on non-informative vision and the perceived position of the hidden hand, with no additional cues derived centrally or through active movement. One could postulate that while pointing provides a measurement of proprioception encompassing both peripheral (i.e. muscle tendons, skin receptors, joint receptor) (or *static*) and central components (i.e. motor commands), the verbal task only captures information from the former (e.g. [3–4,38–41]). It is crucial that the appropriate selection of localisation task is considered in the experimental design of future studies given the observed differences in localisation bias between tasks and their hypothetical measurement of distinct constructs. Further, it is vital that the experimenter ensures no additional proprioceptive cues are derived through any extraneous movement of the hidden hand and fingers.

Overall accuracy improved when the hand was rotated. This could simply reflect the repositioning of the hand as now closer to the body along the transverse plane, hypothetically enhancing one’s ability in determining its location. Indeed, previous studies suggest localisation biases are smaller when the hand is positioned closer to the body [42,43]. With respect to the pointing task, it has been shown that performing a reaching movement towards targets on the same side as the reaching arm (ipsilateral) are faster and more accurate than reaching movements made across the midline (contralateral) of the body [44]. In the current study, all pointing trials made to a straight hand involved reaching across the midline. This was not the case when the hand was rotated.

While we observed an effect for hand tested (dominant vs. non-dominant), the effect was small (1.05 and 1.62 cm along the proximo-distal and radio-ulnar axes respectively). Conclusions as to whether this represents a left-right discrepancy or a difference in hand dominance requires a sample consisting of left-handed participants. Nevertheless, the current findings suggest that the hand tested is of less importance compared to the orientation of the tested hand when performing the landmark localisation task.

### Does the perceived location of the hand drift over time?

Participants in the current study consistently misjudged their hand as *increasingly* closer towards their wrist (proximal drift) throughout each block of trials. No drift was observed along the radio-ulnar axis (apart from a minor drift towards the thumb when during the verbal trials when the right hand was straight). Although the orientation of the hand altered the size of the drift along the radio-ulnar axis, the actual difference in drift between orientations was trivial at 0.69 cm. The fact that proprioceptive drift occurs consistently in the proximal direction independent of hand orientation provides further support for the previous proposition that judgments of perceived hand position are referenced from the hand itself, rather than from the body or external space. Again, this proposition must be interpreted with caution, as we did not precisely standardise the position of the hand and forearm during the rotated conditions.

The actual size of the drift towards the wrist in the current study was notably larger than that previously reported by Wann & Ibrahim [22]. This discrepancy is likely attributable to the relatively brief two-minute timeframe over which Wann & Ibrahim [22] tested each of their participants–substantially shorter than the average 20-minutes taken to complete each block of trials in the current study. Significantly, Wann & Ibrahim [22] showed a linear relationship between drift and time. Therefore, it entirely plausible to suggest the magnitude of the drift towards the wrist may have been comparable between studies had the duration of each been standardised. However, it is highly unlikely that this drift continues indefinitely, and one would anticipate that a steady state would occur at some point. This nevertheless underscores the importance in controlling for the aspect of time when designing future experiments investigating measures of perceived hand location, or alternatively identify the time point at which the drift towards the wrist ceases before commencing the experiment.

### Size and shape of the perceived hand

While the current study replicated the previously reported systematic distortions in hand size and shape [9–14,16–19,36–37], more intriguing were the comparisons of proximal mislocation with perceived finger length, and ulnar mislocation with perceived hand width. This raises the questions as to whether the landmark localisation task actually measures perceived hand *location* or perceived *size and shape*, as is currently accepted. In other words, are the previously reported perceived shortening of fingers and widening of the hand merely an artefact of an overall proximal and (to a lesser extent) ulnar bias, respectively, in perceived hand location? Alternatively, could the proximal bias reported in the current study simply reflect a perceived shortening of finger length? Notably, explicit judgments of hand size and shape using a template-matching task suggest that participants are indeed accurate when selecting the template that most closely resembles the dimensions of their own unseen hand [9,14,15]. As participants are *not* explicitly asked to judge the size and shape of their hand during the landmark localisation task, it seems pre-emptive to infer that the task itself is measuring an internal representation of the dimensions of the hand rather than the simple spatial location of its individual landmarks.

The relationship between ulnar mislocation and hand width is less apparent, given that the former is markedly smaller and less consistent than the latter. A curious proposition by Medina & Duckett [45] suggests that participants tend to increasingly overestimate the distance between successive target landmarks the closer they are to one another. Given the relative proximity of consecutive knuckles compared to that between the knuckles and fingertips, it seems reasonable to propose that the perceived widening of the hand reported both in the current and previous studies is simply a result of participants overestimating the distance between closely approximated landmarks–specifically, the knuckles. Indeed this proposal is supported by the difference in perceived hand width between the pointing and verbal localisation task in the current study, as well as Longo & Haggard’s [14] finding that participants did *not* overestimate the width of their unseen hand when asked to judge whether a visually presented line was shorter or longer than the length they perceived between the knuckles of their index and little fingers–two different tasks which eliminate any potential overestimation bias evoked through pointing. On a separate note, performing the pointing landmark localisation task with the fingers pressed together reduces the amount in which hand width is overestimated by approximately half [12].

A limitation of the verbal localisation task is the lower resolution output of each participant’s landmark judgements, as each selection must fit within a 1 cm x 1 cm superimposed square box. This is more problematic at the proximal landmarks such as the knuckles, where two adjacent landmarks may fall within the same square box. Due to this, participants may have been forced to select the same box despite clearly perceiving a difference in location between the two landmarks. This may have contributed somewhat towards the lack of overestimation of hand width observed in the pointing task and in previous studies. This could be addressed in future studies by using a smaller dimension grid box which would permit greater resolution, allowing participants greater ability to discriminate between landmarks that are anatomically close.

Further work is required to clarify whether participants truly perceive their hand as wider than it actually is. This would be best achieved by a study design that measures both perceived location and perceived size. Indeed, it has been shown that healthy participants overestimate the width of their shoulders and the length of their upper arms relative to their height, but underestimate the length of their forearms and legs [46].

### Clinical implications

Proprioception is compromised in a wide range of neurological disorders, most commonly stroke [47], Parkinson’s disease [48] and multiple sclerosis [49]. Furthermore, there is currently research investigating potential correlations between reduced proprioceptive acuity and recurrent injuries in orthopaedic and sporting populations [50]. And more recently, it has been suggested that impaired proprioception may play an important role in chronic low back pain [51] and whiplash associated disorders [52].

However, when it comes to the clinical assessment of proprioception, a current challenge is to facilitate the translation of research into clinical practice. At present, the methods and devices used in the laboratory are complex, costly and time-consuming–rendering them prohibitive in the clinical setting. Consequently, clinicians continue to use poorly standardised and non-specific tests of proprioception such as ‘standing on one leg with eyes closed’ and the ‘finger to nose’ test (for review, see ref [3–4,53]). Indeed, clinical tests of proprioception fail to discriminate between different sources of proprioceptive information–namely those from peripheral sources (i.e. muscle spindles, joint and skin), centrally-generated motor commands, and higher-level body representations (such as the body model).

A refined version of the landmark localisation task offers potential as a simple, relatively cheap and quick measurement of proprioception. Future experimental designs can use techniques to block proprioceptive input from the periphery, such as an ischaemic block [39], thereby potentially isolating the proposed body model’s contribution to position sense in the hand. Furthermore, the apparatus has scope to be upgraded with the inclusion of force measuring devices, allowing clinicians to quantify the contribution of central motor commands when measuring perceived hand location (e.g. [38,40]). By establishing normative data from healthy populations, researchers will then be able to determine whether or not the body model is implicated in proprioceptive-related movement disorders within specific disease groups.

## Conclusion

The current study reveals that healthy participants consistently misjudge the location of their hidden hand as closer towards their wrist (proximal bias) and, to a lesser extent, away from their thumb (ulnar bias). Furthermore, the perceived location of the hand drifts closer towards the wrist (proximal proprioceptive drift) over time, while remaining stable along the radio-ulnar axis of the hand. Lastly, calculated finger length and hand width–derived from perceived landmark location judgements–reveal shorter fingers and a wider hand compared to the actual hand’s dimensions.

## Supporting information

S1 File The results of each second and first order hierarchical interaction model and each fixed effects only model for each analysis. (XLSX)
